# Multiparametric prenatal imaging characterization of fetal brain edema in Chiari II malformation might help to select candidates for fetal surgery

**DOI:** 10.1007/s00330-024-10729-0

**Published:** 2024-04-24

**Authors:** Hui Shi, Florian Prayer, Patric Kienast, Farjad Khalaveh, Christian Nasel, Julia Binder, Martin. L. Watzenboeck, Michael Weber, Daniela Prayer, Gregor Kasprian

**Affiliations:** 1 Department of Radiology, Zhujiang Hospital, Southern Medical University, No. 253, Industrial Road, Guangzhou, China; 2 Department of Biomedical Imaging and Image-guided Therapy, Medical University of Vienna, Währinger Gürtel 18-20, 1090 Vienna, Austria; 3 Department of Neurosurgery, Medical University of Vienna, Vienna, Austria; 4 Department of Radiology (Diagnostic and Interventional) (C.N.), University Hospital Tulln – Karl Landsteiner Private University of Health Sciences, Alter Ziegelweg 10, 3430 Tulln, Austria; 5 Department of Obstetrics and Feto-maternal Medicine, Medical University of Vienna, Vienna, Austria

**Keywords:** Chiari II malformation, Brain edema, Diffusion tensor imaging, Radiomics, Magnetic resonance imaging

## Abstract

**Objective:**

To identify brain edema in fetuses with Chiari II malformation using a multiparametric approach including structural T2-weighted, diffusion tensor imaging (DTI) metrics, and MRI-based radiomics.

**Methods:**

A single-center retrospective review of MRI scans obtained in fetuses with Chiari II was performed. Brain edema cases were radiologically identified using the following MR criteria: brain parenchymal T2 prolongation, blurring of lamination, and effacement of external CSF spaces. Fractional anisotropy (FA) values were calculated from regions of interest (ROI), including hemispheric parenchyma, internal capsule, and corticospinal tract, and compared group-wise. After 1:1 age matching and manual single-slice 2D segmentation of the fetal brain parenchyma using ITK-Snap, radiomics features were extracted using pyradiomics. Areas under the curve (AUCs) of the features regarding discriminating subgroups were calculated.

**Results:**

Ninety-one fetuses with Chiari II underwent a total of 101 MRI scans at a median gestational age of 24.4 weeks and were included. Fifty scans were visually classified as Chiari II with brain edema group and showed significantly reduced external CSF spaces compared to the nonedema group (9.8 vs. 18.3 mm, *p* < 0.001). FA values of all used ROIs were elevated in the edema group (*p* < 0.001 for all ROIs). The 10 most important radiomics features showed an AUC of 0.81 (95%CI: 0.71, 0.91) for discriminating between Chiari II fetuses with and without edema.

**Conclusions:**

Brain edema in fetuses with Chiari II is common and radiologically detectable on T2-weighted fetal MRI sequences, and DTI-based FA values and radiomics features provide further evidence of microstructure differences between subgroups with and without edema.

**Clinical relevance statement:**

A more severe phenotype of fetuses with Chiari II malformation is characterized by prenatal brain edema and more postnatal clinical morbidity and disability. Fetal brain edema is a promising prenatal MR imaging biomarker candidate for optimizing the risk-benefit evaluation of selection for fetal surgery.

**Key Points:**

- *Brain edema of fetuses prenatally diagnosed with Chiari II malformation is a common, so far unknown, association.*
- *DTI metrics and radiomics confirm microstructural differences between the brains of Chiari II fetuses with and without edema.*
- *Fetal brain edema may explain worse motor outcomes in this Chiari II subgroup, who may substantially benefit from fetal surgery.*

**Graphical Abstract:**

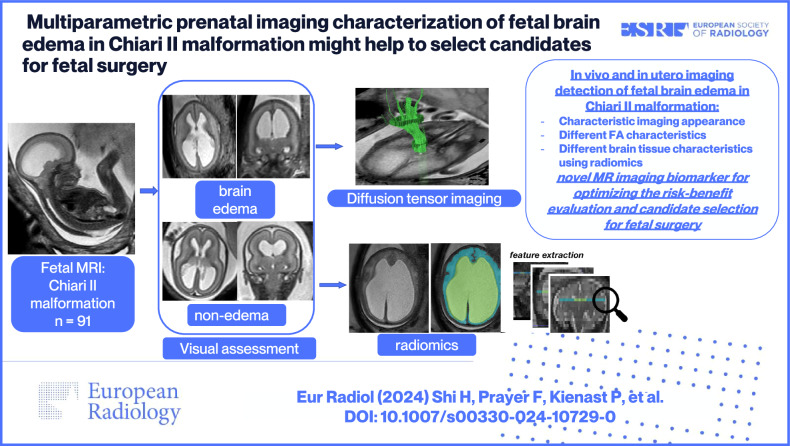

## Introduction

The Chiari II malformation (CM II) describes a congenital downward displacement of the hindbrain through the foramen magnum into the spinal canal, almost always associated with open neural tube defects, such as lumbosacral or thoracic myelomeningoceles (MMCs) [1–3]. Predicting neurological outcomes of this malformation at prenatal stages remains challenging, as “limited” variants of spinal dysraphism have been reported [4], and the postnatal functional level of motor deficits was found to vary across fetuses with the same anatomical defect level at prenatal imaging [2, 5–9]. In 23.6–53% of cases, motor outcomes are worse than what is anatomically anticipated [10]. This indicates the presence of an additional negative impact by the CM II on the fetal brain parenchyma and its motor system—specifically the upper motor neuron and its corticospinal tract (CST) [8, 11]. Further, CM II frequently results in severe and refractory motor and cognitive changes, which persist even after CSF diversion and adequate posterior fossa decompression [2].

Structural correlates being associated with or resulting from CM II comprise white matter volume loss (partially decreased or globally defective) [12], migrational defects resulting in white [13, 14] and gray matter [12, 15] abnormalities frequently involving the motor cortex, and CSTs negatively affecting gross and fine motor skills [6, 11]. Venous congestion due to altered venous return secondary to the small posterior fossa and incomplete expansion of the prosencephalic vesicles secondary to the CSF leak through the open spinal dysraphism are possible etiologies of the supratentorial structural changes [5] and are indicated by subsequent vasogenic brain edema [16, 17].

Prenatal surgery aims to prevent primary damage to neuronal tissue at the level of the defect or secondary neuronal loss as a result of hydrocephalus—and fetal brain edema [18–20]. However, the phenomenon of venous congestion and fetal brain edema has not yet been included in the phenotypic characterization of CM II, which could be used for stratification of severity of supratentorial involvement. Fetal MRI-based identification of fetal brain edema may serve to support and optimize the selection of cases with the biggest benefit of fetal surgery. Thus, this study aimed to initially describe the fetal MR phenotype of fetal brain edema in a retrospective cohort of CM II fetuses by visual radiological assessment, in-utero diffusion tensor imaging (DTI), and radiomics.

## Materials and methods

### Participants

This retrospective single-center study was approved by the institutional internal review board (Ethics Committee number 1716/2017). Some study participants were included in previous studies that did not include brain edema analysis [8, 21, 22].

Inclusion criteria for the study were the presence of CM II—as characterized by the lemon configuration of the fetal head, small posterior fossa, brainstem elongated and kinked, vermis displacement, and reduced width of the external CSF spaces [3, 23–25]. Fetuses (singleton and multiple pregnancies) with these MR findings of CM II present between 1 January 2007 and 31 December 2021 were included in this study. As a reference standard, 1:1 age-matched (+/−5 gestational days) fetuses with structurally normal brain development on ultrasound and MRI were selected from an existing database as healthy controls. The indications for MRI in the control group were detailed in Appendix Table 1.

### Fetal MRI

In vivo fetal MRI was performed according to the ISUOG Practice Guidelines [26] using 1.5-T scanner (Philips Ingenia with a 32-channel body coil; Philips Medical Systems). In each case, routine fetal head sequences T2-weighted turbo spin echo (TSE) (TE: 100 & 140 ms, TR: 2000 ms, acquisition matrix: 256 × 256 voxels, FOV: 200–230 mm, NSA: 1, flip angle 90°) was performed in three perpendicular planes resulting in an in-plane resolution of 0.62–1.0 mm (slice thickness 2.0–4.4 mm). FLAIR imaging and EPI-T2* weighted blood-sensitive sequence were also performed (Appendix Table 2). Specific absorption rate levels did not exceed 2.0 W per kilogram of body weight.

Axial single-shot, fat-suppressed, echo planar DTI sequence (16 noncollinear diffusion gradient-encoding directions with *b*-values of 0 and 700 s/mm^2^, TE 90 ms, TR variable “shortest”, 1457–2130 ms (mean, 1745.31 ms), flip angle 90°, FOV 240, matrix 112 × 105, slice thickness 3 mm, acquisition time 1 min 16 s, perpendicular to the axis of Meynert (long axis of the fetal brainstem), as previously described [22, 27].

### MRI image analysis

#### Structure T2-weighted imaging (T2WI) analysis

**Fig. 1:**
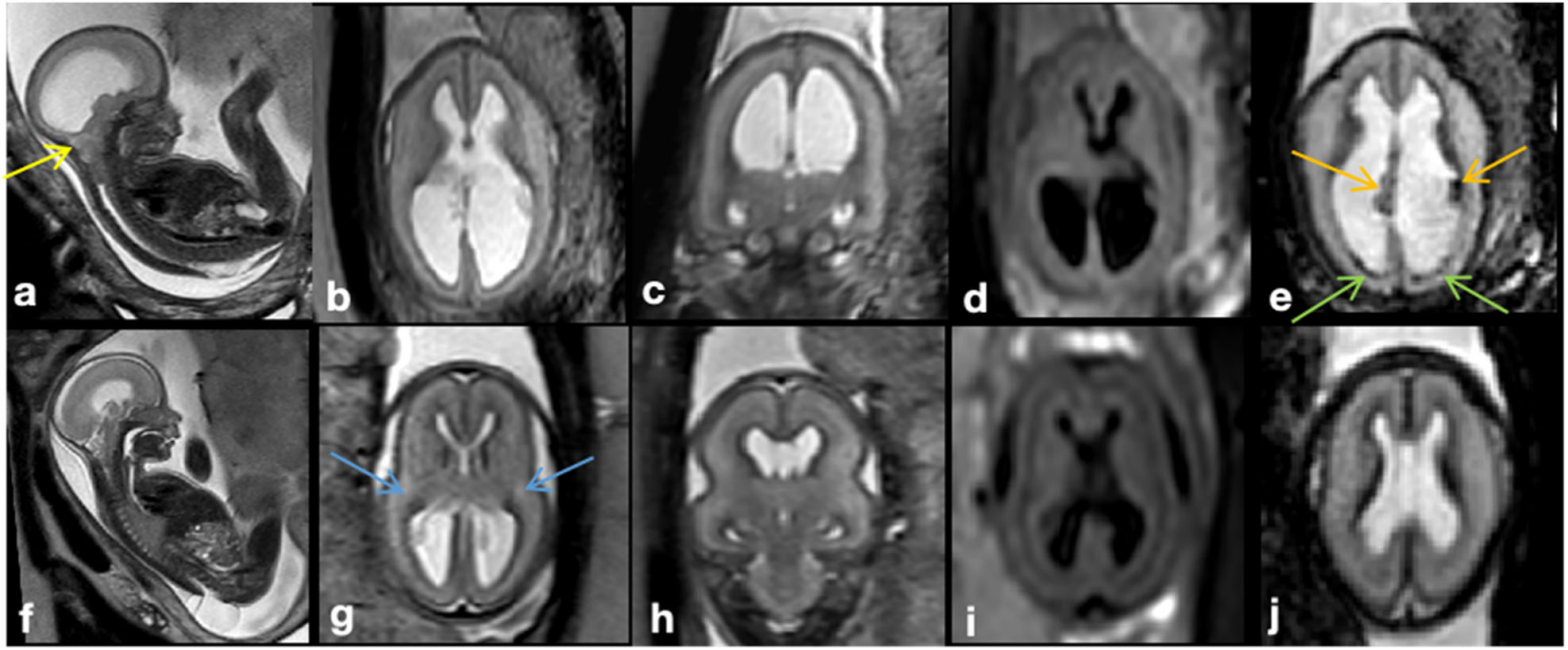
**a–e** MRI of a fetus with Chiari II malformation and brain edema at GW24 + 0, spinal defect formation at the lumbar level (L1/2) extending to the sacral level (S1). The posterior cranial fossa was very crowded and the vermis reached up to about C5, accompanied by protrusion of the atlantooccipital membrane (yellow arrow). **b** T2W-FSE images showed the compressed brain parenchyma with higher signal intensity compared to the age-matched nonedema case (without identifying the parietal crossroads) and effacement of external CSF spaces. **c** Blurring of lamination was shown on T2-Flair sequences. **e** Blood-sensitive sequences showed congested, periventricular veins (orange arrow; see Appendix Fig. 2 for postmortem MRI confirmation) and tiny hemorrhages (green arrow) along the ependyma of the posterior horn of the lateral ventricles. **f**–**j** MRI of a fetus with Chiari II malformation without brain edema at GW24 + 0. A spinal defect was found at the level of S1. **f**–**h** T2W-FSE image showed preserved external CSF spaces and distinguishable hyperintensity of the parietal crossroads in a triangle shape (blue arrows). **i** Normal brain lamination was shown on T2-Flair sequences. **j** Blood-sensitive image was normal

**Fig. 2:**
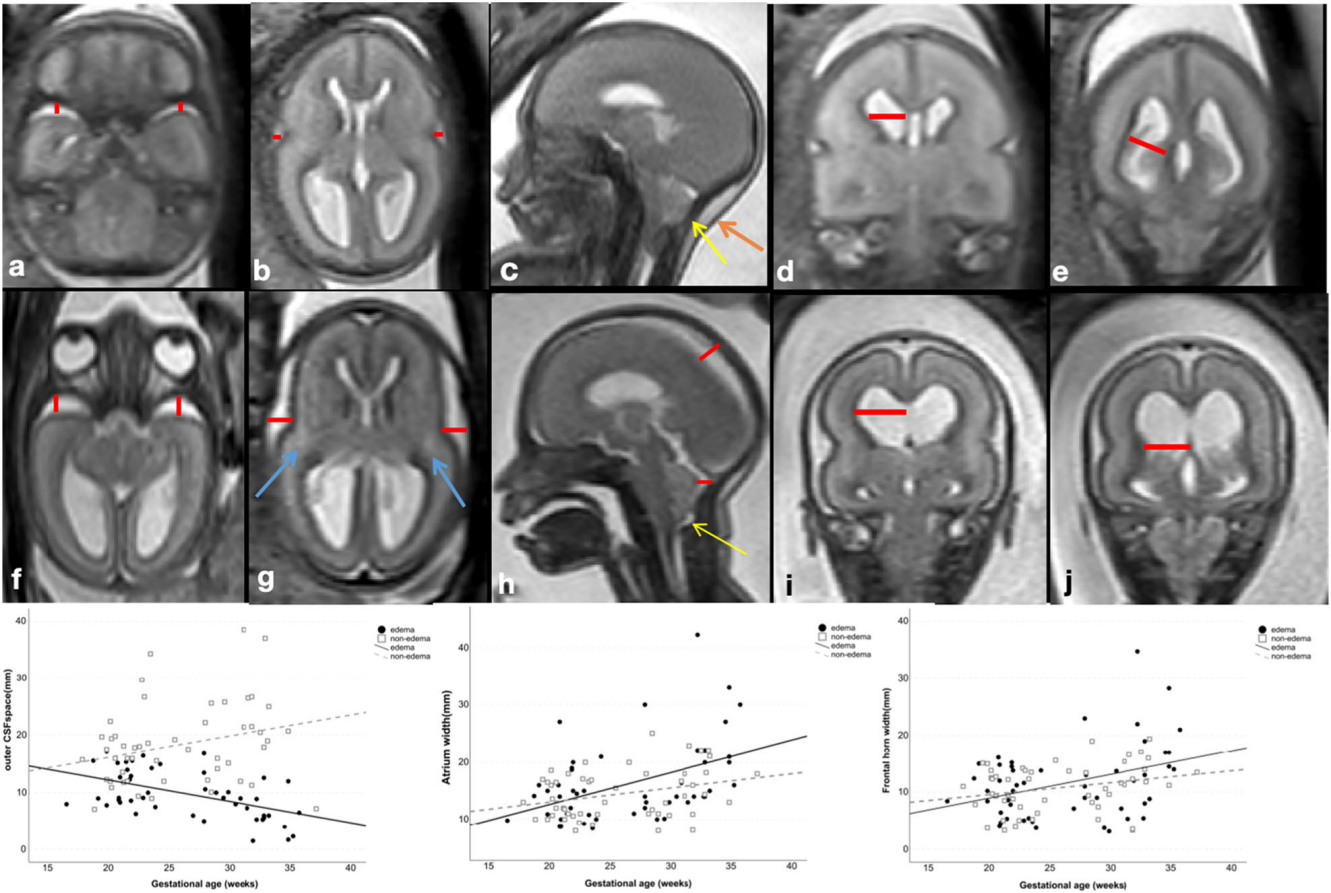
Example of external CSF space and frontal horn/atrium width measurement. **a**–**e** T2-weighted images of a fetus with Chiari II malformation and edema at GW23 + 3. The brain parenchyma was edematous and swollen, with outer cerebrospinal fluid spaces are only detectable in the insular cistern and temporobasal cistern (axial plane), as well as central and retrocerebellar subarachnoid spaces (mid-sagittal plane), and all the above width measurements (red lines) were summarized as total external CSF space for comparison. The frontal horn and atrium width of the lateral ventricle were also measured. Note the protrusion of the atlantooccipital membrane (yellow arrow) and neck edema (orange arrow). **f**–**j** T2-weighted images of an age-matched nonedema fetus at GW23 + 0, external cerebrospinal fluid spaces were preserved, and a triangle-shaped hyperintensity of the parietal crossroads can be 1identified (blue arrows). Protrusion of the atlantooccipital membrane was also presented in this case (yellow arrow). Scatterplot of outer CSF spaces and atrium/frontal horn width throughout the investigated gestational weeks in edema and nonedema groups. A linear decline in outer CSF space width was shown among fetuses investigated in the edema group, with an R^2^ value of 0.261. GA gestational age given in weeks

A systematic analysis of CM II and spinal defect characteristics was conducted [19] and compared between groups. Brain edema cases were retrospectively classified by two experienced fetal neuroradiologists independently (G.K. and H.S., with 15 and 5 years of experience in fetal imaging, respectively) based on the following characteristics: visually apparent brain edema detected by a clearly higher signal intensity on T2-weighted fast spin echo (T2W-FSE) sequences as compared to the age-matched normal brain, blurring of lamination on T2-weighted and/or T2-Flair sequences and effacement of external CSF spaces (Figs. 1 and 2). The readers were blinded to the fetal MRI findings and postnatal outcomes. Disagreements were resolved by consensus. Fetal MRI scans were rerated regarding intrarater variability analysis after 2 months.

#### DTI analysis

**Fig. 3:**
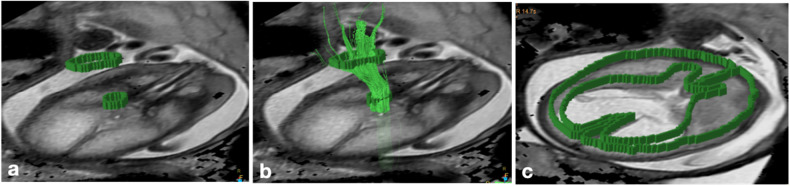
**a**, **b** Characterization of the right corticospinal trajectories by two ROIs, one located at the cerebral peduncles and another at the posterior limb of the internal capsule in a fetus with CM II and brain edema at 28 GW. **c** A single polygonal ROI was placed at the level of the falx delineating the whole brain parenchyma, to calculate brain parenchyma ADC/FA value

The aligned T2-weighted and DTI sequences were post-processed using the Diffusion Registration package of the Philips Achieva workstation (release 2.1.1.0). The corticospinal trajectories were characterized by two polygonal regions of interests (ROIs) on axial slices of the aligned T2-weighted images, one located at the cerebral peduncles and another at the posterior limb of the internal capsule [27]. Fiber tracts were visualized using a deterministic linear tracking algorithm with a fractional anisotropy (FA) threshold of 0.15 and a maximum angle change of 27.0˚ [28]. Tractography was rated as “successful” if the visualized CST fiber morphology corresponded to the 2D axial T2-weighted white matter anatomy. Mean FA and apparent diffusion coefficient (ADC) values were determined only in ROIs of successfully visualized CST fibers to ensure precise measurements. For brain parenchyma ADC and FA value calculation, a single polygonal ROI was placed at the level of the falx (Appendix Fig. 1), delineating the whole brain parenchyma (Fig. 3). Two examiners (G.K. and H.S.) agreed on the delineation process prior to placing the ROIs.

#### Radiomics

**Fig. 4:**
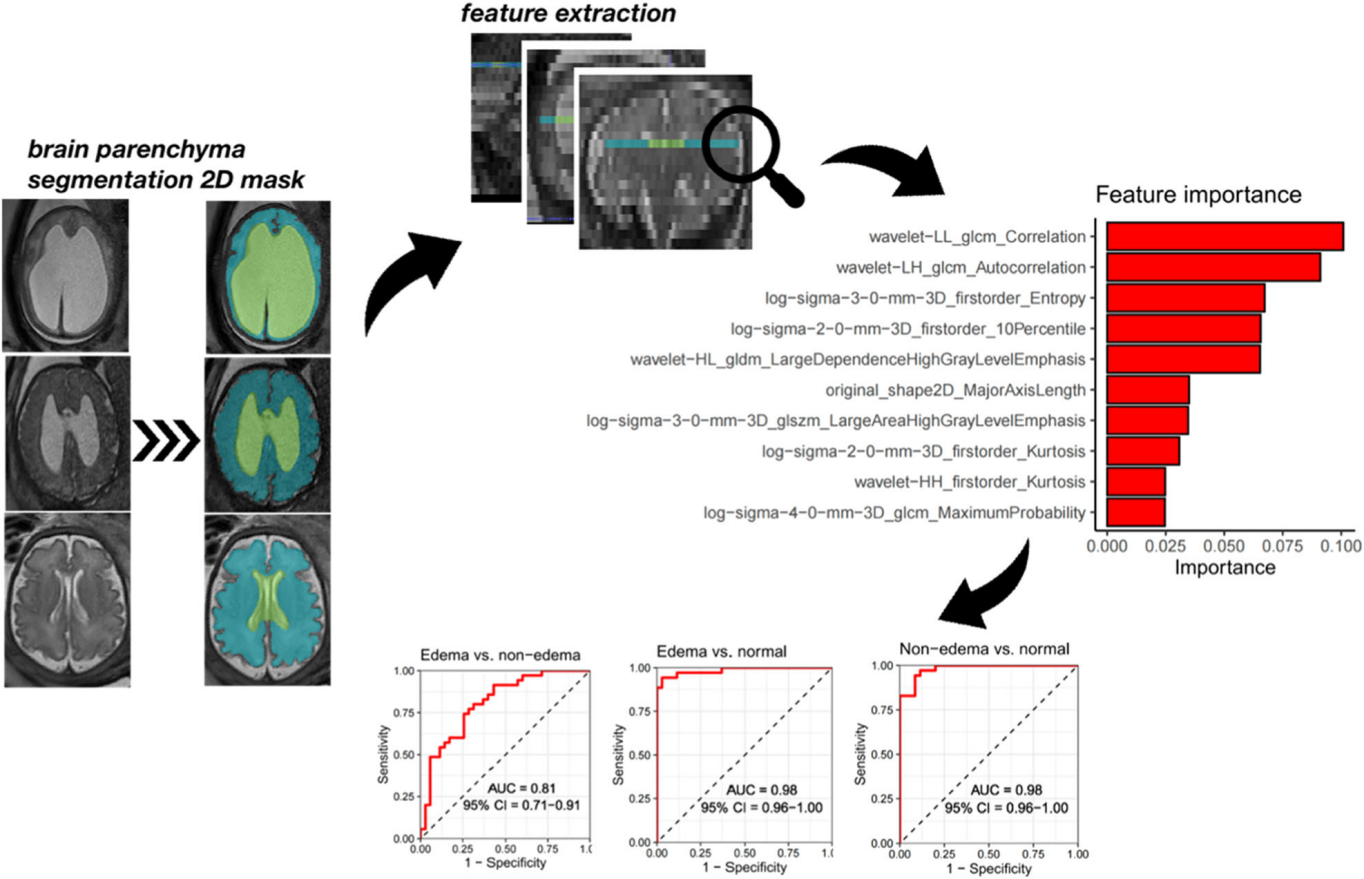
The diagram shows the workflow for extracting radiomics features from T2W-FSE images. Examples of brain parenchyma segmentation two-dimensional (2D) masks were shown in 3 1:1 age-matched representatives in the edema/nonedema/normal group at GW32 + 3,32 + 3,33, respectively. 2D ROIs were defined at the level of the falx, which was the same level as used in DTI hemisphere segmentation. The ten most important features, which were predominantly texture features, were plotted. The ROC curves of the features showed an AUC of 0.81 (95%CI: 0.71,0.91) for discriminating Chiari II fetuses with edema from those without edema and AUCs of 0.98 (95% CI: 0.96, 1 for both) for discriminating Chiari II cases with and without edema from normal controls. GLCM gray level co-occurrence matrix, GLDM gray level dependence matrix, GLSZM gray level size zone matrix

To further validate the concept of the brain edema phenotype in fetuses with CM II and explore hidden microstructure changes accompanying T2WI signal intensity elevation, we performed analysis of radiomics features extracted from brain parenchyma. The axial T2-TSE image data of cases with excellent quality was anonymized and exported from the institute’s PACS (Dedalus HealthCare). Manual segmentation of the brain parenchyma was performed by one radiologist (H.S.) on a single slice at the level of the falx, which was the same level used for DTI hemisphere segmentation. This segmentation was performed using the open-source software ITK-Snap [29]. The resulting brain parenchyma segmentation 2D masks and MRI images were exported as nifti-files, and radiomics features were extracted using the open-source Python package pyradiomics (version 3.0.1.) running under Python 3.7.1 [30] with the following settings: normalise parameter ‘true’, normalise Scale parameter 100, voxelArrayShift 300, (3 SDs × 100) ensuring that only outlier values > 3 SDs below the mean remain negative, binWidth 5, ‘sitkBSpline’ as interpolator, and resampledPixelSpacing ‘[2, 2]’ for 2D image data [31]. Before features extraction, images were transformed using Laplacian of Gaussian filters with sigma values of 2, 3, 4, or 5 mm, and a wavelet filter. A total of 783 radiomics features were extracted using the aforementioned parameters from the original images, as well as the images resulting from the filtering transformations. A gradient boosting classifier, (‘XGBoost’ as implemented in the xgboost python package, version 1.5.0, using default settings), was trained to predict class labels for normal brains, patients with edema and patients without edema, in a binary fashion. Classification accuracy was evaluated using leave-one-out cross-validation, whereby each patient was iteratively left out, the model was trained on the remaining patients, and the class label was predicted for the left-out patient. ROC area under the curve (AUC) metrics were calculated based on the predicted probabilities for the left-out patients in the Leave-One-Out-Cross-Validation framework. To determine feature importances, a final model was trained on all patients after cross-validation. Flowcharts for radiomics feature extraction are shown in Fig. 4.

### Statistics analysis

Statistical analysis was carried out using SPSS Statistics for Windows, v. 25 (IBM Corp., Armonk, NY, USA). Quantitative variables were compared using Student’s t-test for independent samples; qualitative variables were compared using Pearson’s χ^2^. Data Visualization Dimensionality Reduction Analysis/t-distributed Stochastic Neighbor Embedding(t-SNE) was utilized to visualize patterns and clusters in the morphology data of the edema/nonedema groups.

Analysis of covariance (ANCOVA) was used to compare the FA and ADC values between CM II cases with and without edema and normal controls, corrected for gestational age. Cohen k was calculated to assess intra- and interrater agreement regarding the identification of brain edema on fetal MRI scans. Areas under the curve (AUCs) of the radiomics features were calculated. Statistical analysis was performed by two statistical analysts with significant statistical expertise (M.W. and M.M., with 30 and 5 years of experience, respectively).

## Results

### Fetal characteristics and postnatal outcome

Ninety-one fetuses (41 male fetuses) underwent a total of 101 MR scans obtained at a median gestational age of 24.4 weeks (interquartile range, 10.1 weeks) were confirmed as MMC by postnatal defect repair surgery or postmortem examination and met the study inclusion criteria (see Table 1). Twenty-one MR scans were excluded for cases lost to follow-up. Twelve cases were excluded from the study due to additional congenital malformation or inadequate image quality.

**Table 1:** Fetal demographics and MRI characteristics

Characteristics	All fetuses (*n* = 91)
Fetal characteristics	
Number of fetuses	91
Male fetuses (%)	41/91 (45.1)
MRI	
Number of MRI scans	101
Median gestational age at fetal MRI	24.4 (16.7–37.3)
Number of fetal MRI scans	
1	83
2	6
3	2
Incidence of brain edema (%)	43/91 (47.2)

**Fig. 5:**
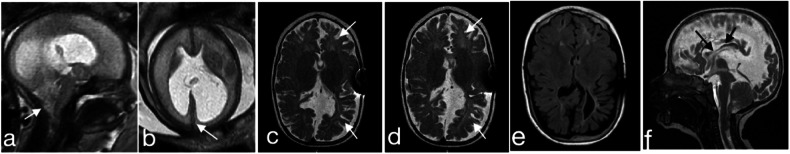
Postnatal follow-up MRI of a Chiari II malformation with brain edema case. **a** Prenatal MRI performed at 27 + 3 weeks, showed small posterior fossa, severe vermis ectopia, kinking of the brainstem, and protrusion of the atlantooccipital membrane (white arrow). **b** Axial plane showed diffuse brain parenchyma edema and compressed superior sagittal sinus (white arrows). **c**–**e** Global white matter volume loss and gliosis were shown on postnatal follow-up MRI after shunting conducted at 9 months (**c**) and 3 years of age (**d**, **e**), respectively. **f** The sagittal plane showed misshaped corpus callosum as a consequence of prenatal compression on the one hand and postnatal shunt implantation on the other hand (black arrows)

Follow-up time ranges between 5 days to 16 years. Fourty-eight cases were terminations of pregnancy. Of the 43 cases that received in-utero or postnatal repair surgery, 35 patients (81.4%) underwent postnatal MRI. Residual edema was still evident on the postnatal MRI performed within the first month of birth (see Appendix Fig. 2). A higher prevalence of white matter volume loss (18/20 vs. 5/15, Fig. 5), and intracranial hemorrhage was notable (6/20 vs. 0/15) in the edema group (Appendix Fig. and Table 3). In the edema group, 18/23 cases, and in the nonedema group 10/20 cases received CSF shunting. Poor outcome events (a worse functional level than anatomically expected) occurred in 6/23 (26%) cases in the edema group, while the functional and anatomical levels matched postnatally in all 20 cases without any instances of a lower functional level in the nonedema group.

### MRI image analysis

#### Structure T2WI analysis

Of the 101 MR scans collected, 50 scans met the phenotypic criteria of fetal brain edema, while the remaining 51 scans showed preservation of outer CSF spaces and clear brain lamination (Fig. 1) and were classified as without edema. Intra-and interrater agreement regarding the classification of edema and nonedema was *k* = 0.86 (*p* < 0.001) and *k* = 0.82 (*p* < 0.001), respectively.

**Fig. 6:**
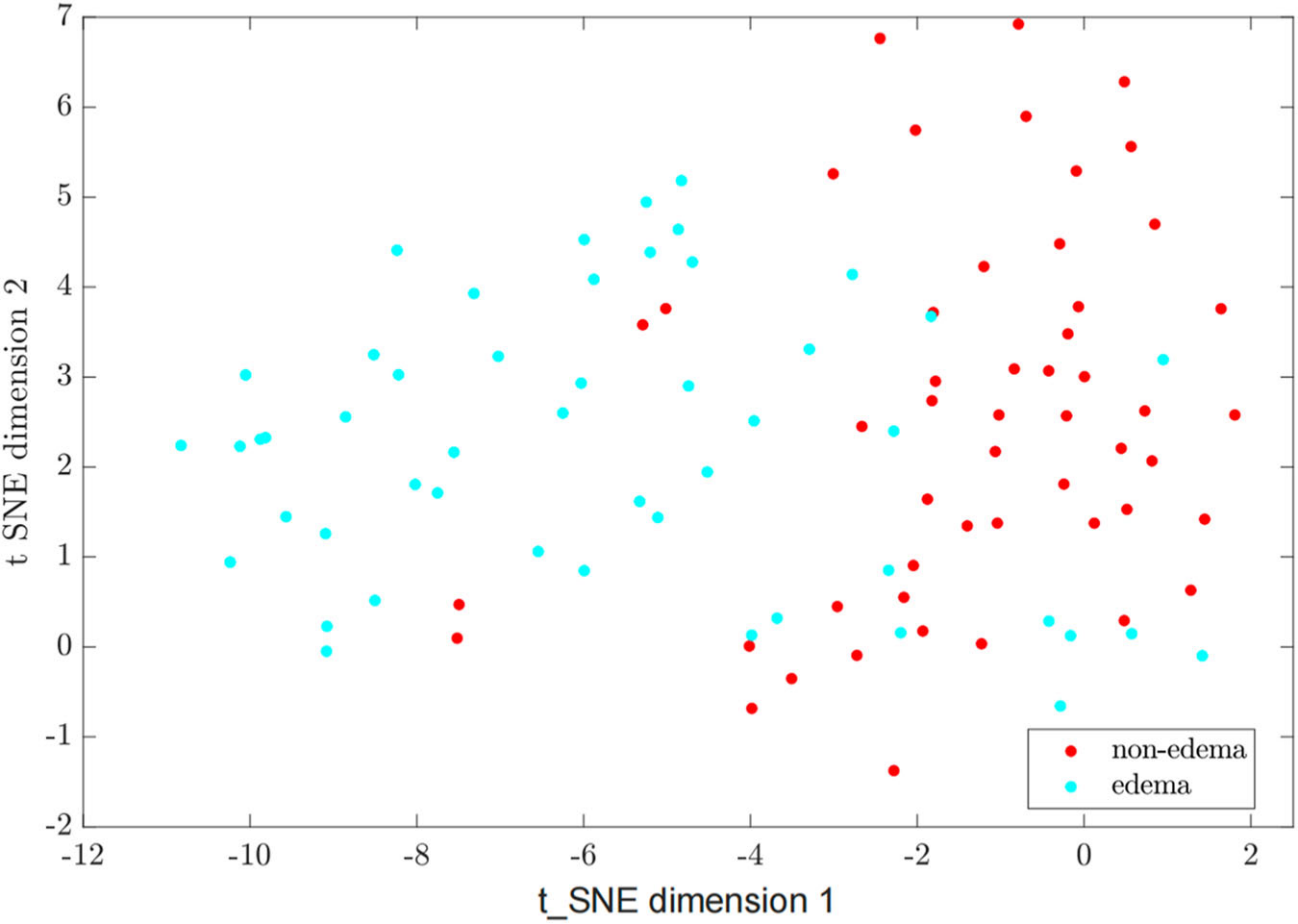
Two-dimensional t-SNE analysis revealed distinct clustering of the edema and nonedema groups based on the Chiari II malformation-associated features

Measurements of the external CSF spaces, frontal horn and atrium width of the lateral ventricle, incidence of hydrocephalus, upper level and size of the spinal defect, and vermian displacement with or without protrusion of atlantooccipital membrane of fetuses with and without edema are summarized in Table 2. After adjusting for the effect of gestational age (GA) using analysis of covariance, groups differed in the size of the outer CSF space (9.8 ± 0.83 mm in the edema group, 18.3 ± 0.82 mm in the nonedema group, *p* < 0.001). We observed a linear decline, while GA was increasing in the outer CSF spaces among all fetuses investigated in the edema group (as illustrated in Fig. 4). Additionally, larger defect size, deeper vermian displacement, higher incidence of hydrocephalus and the protrusion sign of the atlantooccipital membrane were found in the edema group (Table 2). Two-dimensional t-SNE analysis revealed distinct clustering of the edema and nonedema groups based on these CM II-associated features which showed significant *p* values (Fig. 6).

**Table 2:** Fetal and MRI structure characteristics comparisons between with and without edema groups

Parameters	Edema group (*n* = 50)	Nonedema group (*n* = 51)	p*
Male fetuses (%)	42 (21/50)	49 (25/51)	0.479
Median gestational age at fetal MRI (weeks)	25.7 (16.7–35.9)	24.1 (18–37.3)	0.338
External CSF spaces (mm)	9.8^a^ ± 0.83	18.3^a^ ± 0.82	< 0.001
Atrium width (mm)	16.1^a^ ± 0.76	14.6^a^ ± 0.75	0.165
Frontal horn width (mm)	11.5^a^ ± 0.73	10.9^a^ ± 0.73	0.519
Incidence of hydrocephalus (%)	82 (41/50)	62.7 (32/51)	0.031
Anatomic level (%)			
Sacral	26 (13/50)	37 (19/51)	0.224
Lumbar	62 (31/50)	51 (26/51)	0.264
Thoracic	12 (6/50)	11.8 (6/51)	0.971
Size of defect (mm)	27.0	18.1	0.001
Vermian displacement (mm)	13.5	6.4	< 0.001
Protrusion sign of atlantooccipital memberane (%)	92 (46/50)	43 (22/51)	< 0.001

Data are given as median (range), *n* (%) or mean ± SD. *Comparison between with and without edema groups: quantitative variables compared using Student’s *t*-test for independent samples; qualitative variables compared using Pearson’s χ^2^

^a^ Estimated means based on an average GA of: GA = 26.3

#### DTI analysis

Out of 101 MR scans, 71 were excluded due to either the absence of DTI, inadequate DTI quality, or unavailability of age matching. The remaining 30 age-matched MR scans (15 with, 15 without edema) and 15 1:1 age-matched control cases were included in the DTI analysis.

To control for the effect of GA, an analysis of covariance was used (Table 3). FA values decreased across the groups, with the edema group demonstrating the highest values and the control group showing the lowest values. However, we found no significant difference in ADC values between the groups.

**Table 3:** FA/ADC values in 3 ROIs comparisons between Chiari II malformation with and without edema and normal control groups

Value	Controls (*n* = 15)		edema (*n* = 15)		Nonedema (*n* = 15)		p*
	Mean	SD	Mean	SD	Mean	SD	
CST FA	0.270	0.013	0.416	0.021	0.340	0.018	< 0.001
Internal capsule FA	0.221	0.015	0.377	0.027	0.281	0.024	< 0.001
Hemisphere FA	0.223	0.015	0.403	0.029	0.293	0.024	< 0.001
CST ADC (10^-3^mm^2^/s)	1.487	0.035	1.633	0.047	1.515	0.058	0.103
Internal capsule ADC (10^-3^mm^2^/s)	1.245	0.033	1.339	0.048	1.250	0.055	0.221
Hemisphere ADC (10^-3^mm^2^/s)	1.508	0.023	1.459	0.065	1.446	0.050	0.571

Data are given as mean ± SD for an average gestational age of 26.3 weeks. Because the right and left values were not significantly different, these values were averaged for analysis. *Effect of gestational age on parameters was eliminated using analysis of covariance. *SD* standard deviations

#### Radiomics

After excluding 31 cases due to the unavailability of age matching, 70 age-matched MR scans (35 with, 35 without edema), and 35 1:1 age-matched fetuses with normal brains were included in the radiomics analysis.

The resulting 10 most important features extracted from the brain parenchyma are shown in Table 4, Fig. 5. These features were predominantly texture features, with 3 belonging to the class GLCM (gray level co-occurrence matrix), 4 belonging to the class ‘First Order’, and the remaining 3 belonging to the class of GLDM (gray level dependence matrix), Shape, and GLSZM (gray level size zone matrix), respectively. The ROC curves of these features can discriminate Chiari II fetuses with edema from those without edema with an AUC of 0.81 (95%CI: 0.71, 0.91) and Chiari II cases with and without edema from normal controls with an AUC of 0.98 (95% CI: 0.96, 1 for both).

**Table 4:** The top 10 important radiomics features

Radiomics features	Classification	Classification	Importance	Mean ± SD	
				Edema	Nonedema
Correlation	Texture features	GLCM	0.1	0.74 ± 0.12	0.62 + 0.12
Autocorrelation	Texture features	GLCM	0.092	352 ± 29	594 ± 49
Entropy	First order statistics	Log-sigma-3-0-mm-3D	0.071	3.88 ± 0.4	4.06 ± 0.3
10 Percentile	First order statistics	Log-sigma-2-0-mm-3D	0.068	-20 ± 6.22	-15 ± 3.18
Large dependence high gray level emphasis	Texture features	GLDM	0.067	1257 ± 106	1754 ± 180
Major axis length	Shape 2D	Original	0.041	104 ± 34	83 ± 19
Large area high gray level emphasis	Texture features	GLSZM	0.041	1108 ± 73	679 ± 41
Kurtosis	First order statistics	Log-sigma-2-0-mm-3D	0.037	3.6 ± 1.0	3.1 ± 0.7
Kurtosis	First order statistics	Wavelet-HH	0.025	5.4 ± 2.3	5.0 ± 1.6
Maximum probability	Texture features	GLCM	0.025	0.04 ± 0.01	0.03 ± 0.01

Top 10 important radiomics features discriminating Chiari II malformation subgroups between with or without edema and normal controls. *GLCM* gray level co-occurrence matrix, *GLDM* gray level dependence matrix, *GLSZM* gray level size zone matrix, *SD* standard deviations

## Discussion

Fetal brain edema in CM II cases—most likely resulting from venous congestion and abnormal CSF circulation—can be detected and objectified by fetal brain MRI. In this study, edematous changes were identified in 43/91 cases following fetal MR imaging criteria. Using the same slice level for brain parenchyma segmentation, both DTI-based FA values and radiomics features microstructural changes in the edema group could be verified. Consequently, this study suggested that fetuses with neural tube defects and CM II malformation may be subdivided into radiological phenotypic subgroups—one being associated with and the other without fetal brain edema.

Fetal brain edema in CM II can be identified by radiological assessment using the following criteria (Figs. 1 and 2): depleted outer CSF spaces, global hyperintense brain parenchyma on T2-weighted images (compared to the age-matched normal brain), and blurred laminar compartmental organization of the fetal brain. Except for the shrinking of outer CSF spaces as a hallmark, 92% of cases with fetal brain edema (vs. 43% of cases in the nonedema group) showed a protrusion of the atlantooccipital membrane associated with severe vermian displacement and kinking of the medulla oblongata, which may result in partial obstruction of venous return (Appendix Fig. 4) that can cause tissue edema. The proliferation of the capillary network also can be impaired in developing brain tissue. Both ischaemic and hemorrhagic infarcts may occur (Fig. 5 and Appendix Fig. 3). When judging the signal intensity of brain parenchyma, pronounced T2-weighted hyperintensity of the parietal crossroads [32] may additionally be helpful in the identification of these cases (Figs. 1 and 2).

Fetal brain edema in CM II may impact cortical formation, neuronal migration, and postmigrational development, specifically affecting the upper motor neurons and their CSTs [33]. While edematous brain change can be detected by structural fetal MRI, DTI-based FA values provide evidence for disruption of tissue microstructure, including axons and myelin in white matter tracts extending beyond structure changes [34]. To enhance the reliability of FA values in assessing brain edema, a deterministic linear tracking algorithm was utilized to reduce the influence of multiple sources of in-utero artifacts. Our results indicate that FA values were elevated significantly in the CST, internal capsule region, and hemisphere of the edema group compared to nonedema and normal control groups. Higher FA can be explained by parenchymal compression in the axial plane and suggests that edema in CM II may compromise the integrity of the CST fibers. In contrast, ADC values were insensitive in the detection of microstructural differences between the groups. Higher FA values in the edema subgroup suggest the presence of vasogenic (and not cytotoxic) edema, most likely resulting from venous congestion. This may be a transient effect—only detectable during intrauterine development and a short time after birth (Appendix Fig. 2)—or may even contribute to a certain degree of parenchymal brain damage as present in follow-up postnatal brain MRI examinations (see Appendix Table and Fig. 3 for more details).

Despite the high interrater consistency (kappa = 0.82) in detecting cases with cerebral edema using traditional T2 sequences, subjective rating depends on a variety of factors, such as the level of radiological expertise and MR signal inhomogeneities due to variable distances of the fetal brain from the center of the coil. Here we chose to quantify DTI-based metrics and fetal MR-based radiomics to overcome these limitations in the phenotypic characterization of this specific CM II subgroup. Radiomics is a technique used to extract numerous features that reflect various aspects of shape and texture from 2D or 3D image ROIs [31]. Fetal MRI is proven to be well-suited for the extraction of quantitative fetal lung parenchyma radiomics features, as the image acquisition follows a standardized protocol [35]. In this study, the second-order features (GLCM, GLDM, and GLSZM), which are predominantly texture features that reflect relevant but visually imperceptible tissue characteristics, are identified as the most important features, indicated microstructure changes accompanying the T2-prolongation signal intensity elevation. The ROC curves of the features showed high AUCs, indicating the good performance of fetal MR-based radiomics in distinguishing brain edema from nonedema subgroups.

All classifiers used to visually determine brain edema in this study are at least suggestive of remodeling of telencephalic neuronal fiber- and microvascular networks, indicated by high T2w-signal, DTI-FA, and the most important radiomics features, which could be explained by mechanisms known from adult chronic occlusive hydrocephalus. A progressive blockage at the craniocervical junction in CM II seems qualified to reduce CSF outflow from the ventricles. As the cerebral transmantle pressure gradient increases, the external CSF spaces shrink. Both mechanisms are known to hinder venous drainage leading to venous congestion and dilatation [5, 36], where mostly the paraventricular veins could be affected, as fetal microcirculation may lack full development of the cortical venous drainage [37] (see Appendix Fig. 5). These assumptions are strongly supported by our results, which demonstrate a significant reduction of the external CSF spaces together with signs of paraventricular venous congestion in the CM II edema group. Conceivably, like in adult hydrocephalus, the concomitant loss of cerebral compliance would trigger progressive parenchymal damage, which likewise could be treated by fetal surgery.

According to McLone and Knepper’s Unified Theory of CM II, both open neural tube defects and incomplete spinal occlusion allow for CSF loss to the amniotic fluid [38]. The edema subgroup was specifically characterized by an almost complete absence of the outer CSF spaces except for the temporobasal and insular cisterns, which declined in width during pregnancy despite a trending increase in the width of the atrium/frontal horn of the lateral ventricles/inner CSF spaces. The absence of appropriate CSF circulation surrounding the fetal brain may further impair the equilibrium of CSF production and resorption and further contribute to the genesis of vasogenic edema in these cases. The data presented in this study further support the concept of prenatal surgery—which leads to an expansion of outer CSF spaces and presumably re-establishment of proper CSF dynamics [39, 40] (see Appendix Fig. 6). Future studies will need to demonstrate whether the resolution of fetal brain edema is an early prognostic marker for successful fetal surgery in MMC cases. Further, it needs to be established if the brain edema subgroup of CM II cases may specifically benefit from fetal surgery—indicating that the presence of brain edema in CM II should be considered as an additional future MR selection criterion for prenatal MMC repair.

Our study has some limitations, which most likely do not interfere with the reported main observation of fetal brain edema in Chiari II. First, 48 cases were pregnancy terminations, so we cannot fully assess the ultimate impact of these changes. Second, DTI and radiomics comparisons between fetuses with and without edema and normal controls can only be made on an age-matched basis owning to the developing fetal brain. This leads to a small sample size, which is a general challenge in this field of research. Third, the nonlinear model we used in radiomics has certain limitations, including its hyperparameters and increased model complexity, but it was good enough to prove differences consistently and independently from other approaches –DTI and radiological evaluation, which meets the purpose of this study. Fourth, this study initially identified a specific subgroup of CM II cases retrospectively, and only three fetuses underwent prenatal repair surgery despite being in a long-term longitudinal cohort. Future prospective multicenter studies in a larger number of CM II cases—optimally undergoing fetal surgery—are needed to further understand the clinical impact of the presented radiological subgroup definition.

In conclusion, our study provides evidence that fetal MRI can identify a subgroup of CM II fetuses, showing vasogenic brain edema - most likely due to abnormal CSF production and resorption dynamics. These changes might be suspected visually on T2-weighted sequences but its proof demands the use of DTI-based FA values and radiomic analysis, which allows the quantification of such changes. The fetal MRI-based identification of fetal brain edema in CM II may serve as a potential MR biomarker indicative of the severity of supratentorial involvement.

## Supplementary information

Supplementary Material

## Abbreviations

ADC: Apparent diffusion coefficient
AUC: Area under the curve
CM II: Chiari II malformation
CST: Corticospinal tract
DTI: Diffusion tensor imaging
FA: Fractional anisotropy
MMC: Myelomeningocele
ROI: Regions of interest
