# Supplementary material for: Multiparametric prenatal imaging characterization of fetal brain edema in Chiari II malformation might help to select candidates for fetal surgery
Source: Eur Radiol. 2024 Apr 24;34(10):6384–95. doi: 10.1007/s00330-024-10729-0 (PMC11399183; doi:10.1007/s00330-024-10729-0)
Supplement: Supplementary file 1 — Supplementary Material [file 330_2024_10729_MOESM1_ESM.pdf]

Multiparametric prenatal imaging characterization of fetal brain edema in Chiari II malformation might help to select candidates for fetal surgery

ELECTRONIC SUPPLEMENTARY MATERIAL

Table 1: Referral diagnoses of 50 control cases made by ultrasound

Suspicious diagnosis in ultrasound	n	Prenatal MRI diagnosis
No detected abnormalities on US, but complex malformation syndromes known in siblings	39	No detected abnormalities in fetal or extrafetal structures
Ovary cyst or myoma	11	Confirmed diagnosis

Table 2: Acquisition parameters of FLAIR and EPI-T2* sequence

Sequence	FOV	matrix	Slice thickness /Gap(mm)	Reconstructed Voxel Size(mm)	TR (ms)	TE (ms)	Flip angle	NSA
**FLAIR**	240	192x68	4.0/0.4.0	1.02/1.02/4.00	7000	140	90°	2
**EPI-T2***	230	160x95	4.0	0.90/0.90/4.0	3000	53	90°	2

Table 3: Summary of follow-up postnatal MR abnormalities in the forebrain of 35 Chiari II malformations

Abnormality	Edema group (n=20)	Non-edema group (n=15)
Callosal dysgenesis	9	4
Subependymal heterotopia	8	5
Polymicrogyria/Stenogyri cortex	3	1
Large massa intermedia	19	13
White matter volume loss(from the margin of atrium to the cortex surface <25mm as defective(1))	18	5
Intracranial hemorrhage	6	0

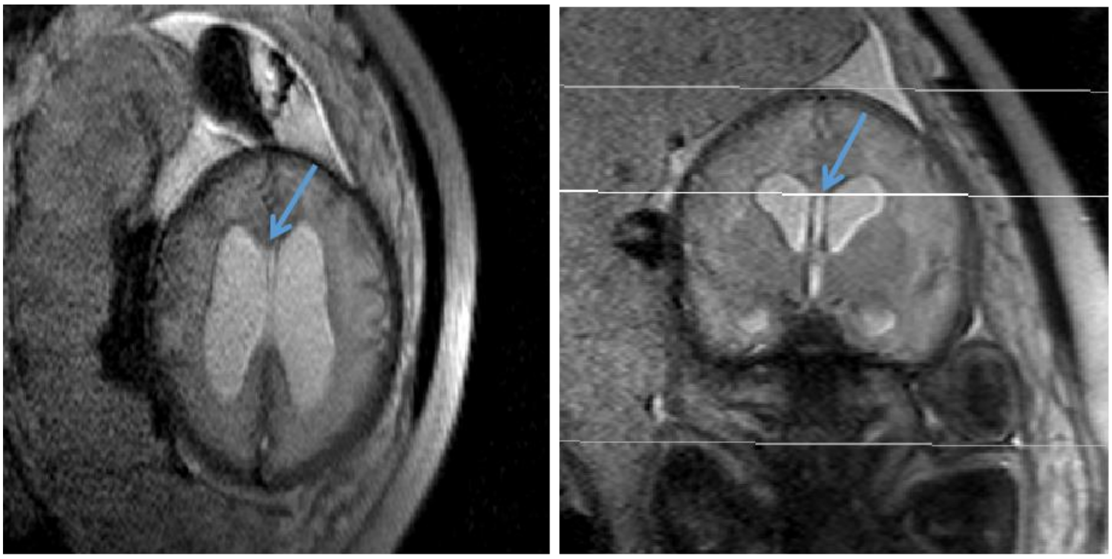

Figure 1: Example of slice level used for DTI and radiomics segmentation.

The slice level on the axial plane matches the falx (arrow) at the top of the septum pellucidum level shown on the coronal plane (see the localization line).

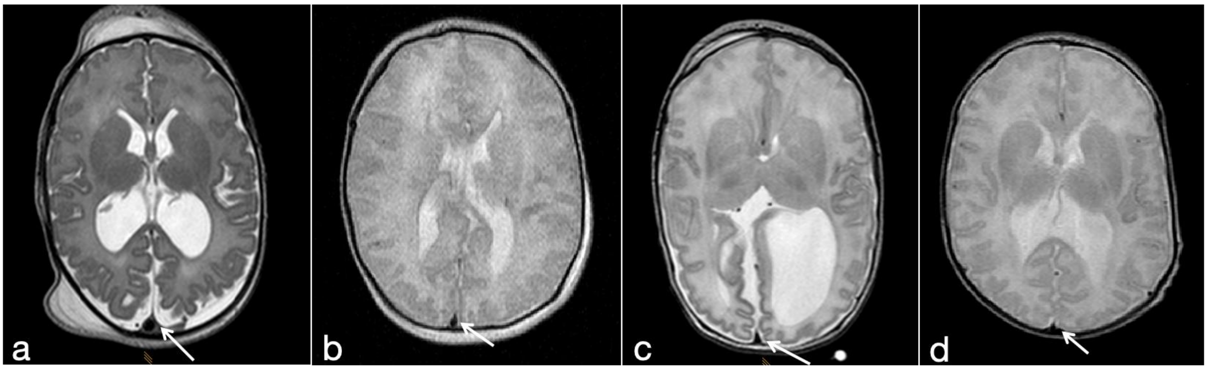

Figure 2: Follow-up postnatal MRI of fetuses with Chiari II malformation and brain edema compared to a meningocystocele case.

(a) Follow-up postnatal MRI of a meningocystocele case performed 5 days after birth showed normal gray and white matter. (b-d) Subsequent postnatal MRI scans of three fetuses with Chiari II malformation and brain edema, conducted at 1 day (b), 6 days (c), and 2 weeks (d), reveal global brain edema characterized by increased white matter intensity, a blurred gray-white matter transition, and reduced outer CSF spaces. Note the compressed superior sagittal sinus(arrows) in the CM II cases compared to the meningocystocele case(a).

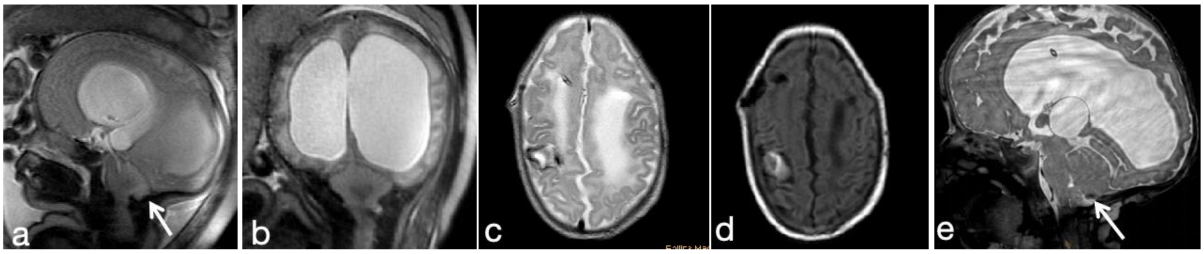

Figure 3: Follow-up postnatal MRI of fetuses with Chiari II malformation and brain edema showed intracranial hemorrhage.

(a, b) Prenatal MRI showed pronounced edema at GW34+0. (b, c) Postnatal MRI was conducted seven weeks after birth, revealing the presence of intracranial hemorrhages in the right post-central parenchyma and the vermis (arrow).

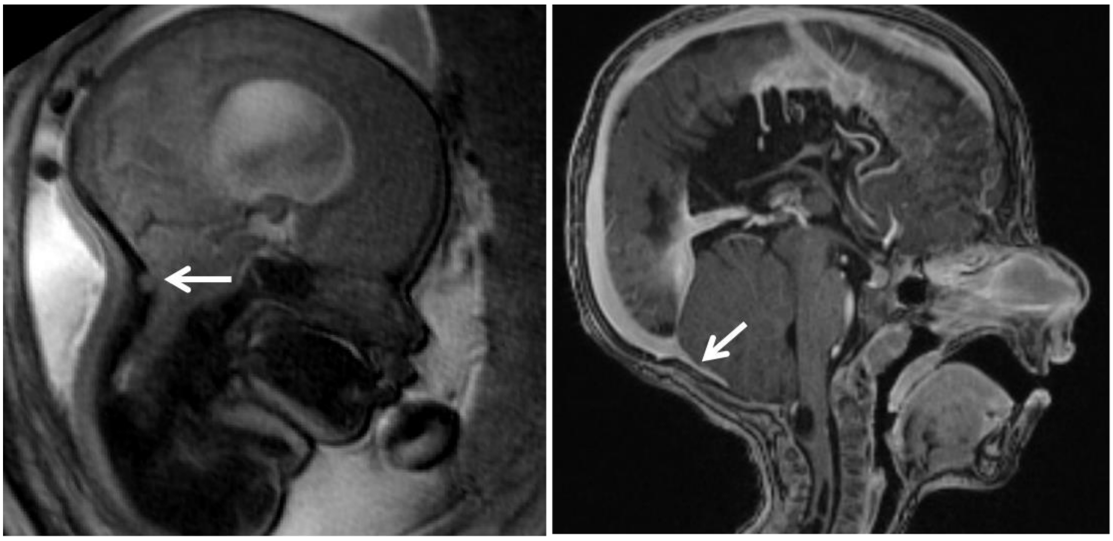

Figure 4: Protrusion of the atlantooccipital membrane showed in a fetus with Chiari II malformation.

The posterior and downward protrusion of the vermis (indicated by the white arrow) creates a concavity in the atlantooccipital membrane, resembling a notch just below the level of the torcula. Postnatal contrast image showed compressed hindbrain vascular structures (especially transverse sinus, white arrow).

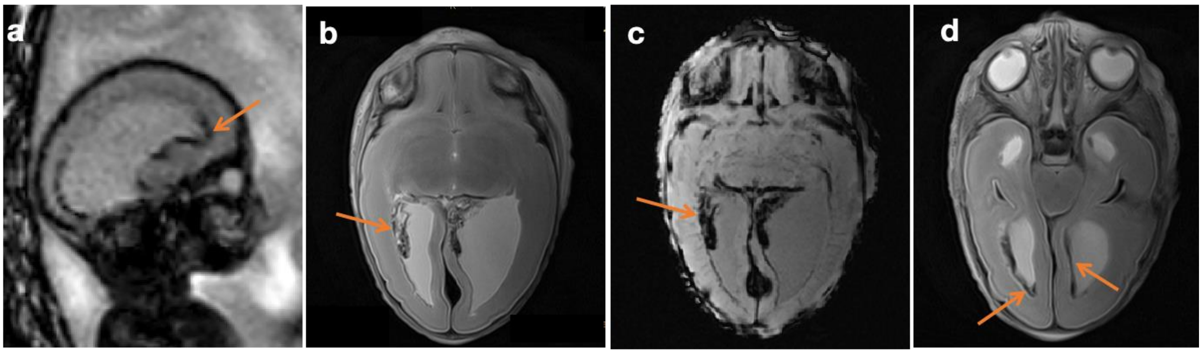

Figure 5: Postmortem MR of a Chiari II malformation with brain edema fetus

(a) Fetal MRI of a Chiari II malformation with brain edema case at 24GW (illustrated in Figure 2 of the main text) showed congested periventricular veins, which were also present on postmortem T2WI and SWI sequences (b, c). (d) In contrast, intra-ventricle hemorrhage was shown with blood-fluid levels in this slice.

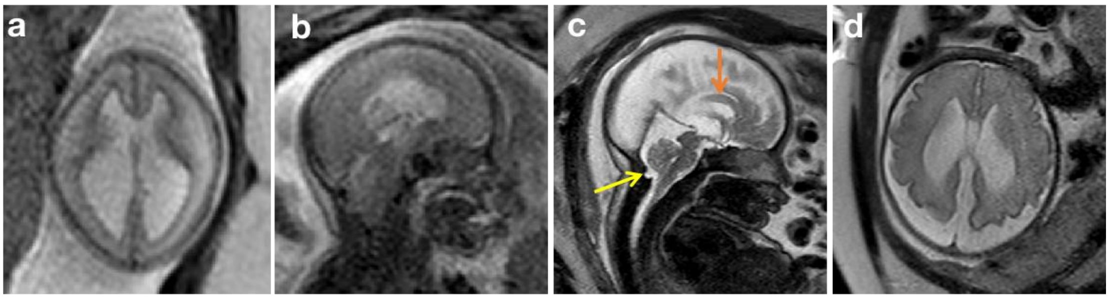

Figure 6: Expansion of Outer CSF spaces after prenatal surgery

(a, b) MRI of a fetus with Chiari II malformation and brain edema at 21GW, showing almost depleted outer CSF space.

(c, d) A follow-up fetal MR exam was performed at 31+4GW after in-utero surgery, which led to an expansion of outer CSF spaces and presumably reestablishment of proper CSF dynamics, and consequently, improved brain edema, noticing the dysgenesis of corpus callosal without splenium part (orange arrow) and the protrusion of the atlantooccipital membrane still present (yellow arrow).

1. Miller E, Widjaja E, Blaser S, Dennis M, Raybaud C. The old and the new: supratentorial MR findings in Chiari II malformation. Childs Nerv Syst. 2008;24(5):563-75.
